# Supplementary material for: Mechanism of AAA+ ATPase-mediated RuvAB–Holliday junction branch migration
Source: Nature. 2022 Aug 24;609(7927):630–9. doi: 10.1038/s41586-022-05121-1 (PMC9477746; doi:10.1038/s41586-022-05121-1)
Supplement: Supplementary file 1 — This file contains Supplementary Fig. 1, Supplementary Tables 1–3 and Supplementary Video legends [file 41586_2022_5121_MOESM1_ESM.pdf]

nature portfolio

https://doi.org/10.1038/s41586-022-05121-1

Supplementary information

Mechanism of AAA+ ATPase-mediated RuvAB–Holliday junction branch migration

In the format provided by the authors and unedited

Mechanism of AAA+ ATPase-mediated RuvAB-Holliday junction branch migration

Jiri Wald[1,2,3,4,5]*, Dirk Fahrenkamp[1,2,3]*, Nikolaus Goessweiner-Mohr[4,5], Wolfgang Lugmayr[1,2,3,4,5], Luciano Ciccarelli[4,5], Oliver Vesper[1,2,3,4,5], and Thomas C. Marlovits[1,2,3,4,5 # *]

[1] Centre for Structural Systems Biology, Notkestraße 85, 22607 Hamburg, Germany

[2] Institute of Structural and Systems Biology, University Medical Center Hamburg-Eppendorf, Notkestraße 85, 22607 Hamburg, Germany

[3] Deutsches Elektronen Synchrotron (DESY), Notkestraße 85, 22607 Hamburg, Germany

[4] Institute of Molecular Biotechnology GmbH (IMBA), Austrian Academy of Sciences, Dr. Bohr-Gasse 5, A-1030 Vienna, Austria

[5] Research Institute of Molecular Pathology (IMP), Campus-Vienna-Biocenter 1, A-1030, Vienna, Austria

* Corresponding authors

[#] Lead corresponding author

Table of content

1) Supplementary information Fig. 1 – Source data
2) Supplementary information Table 1
3) Supplementary information Table 2
4) Supplementary information Table 3
5) Supplementary Video legends (1 – 6)
6) Supplementary Video legends (7 – 11)

Supplementary Fig. 1
Source data

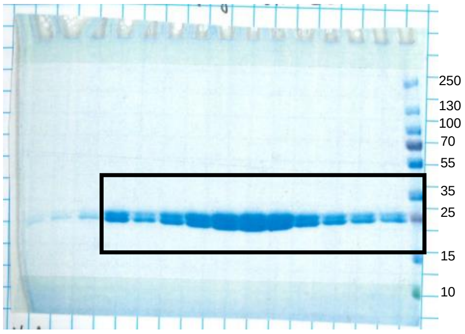

Extended Data Fig. 1a

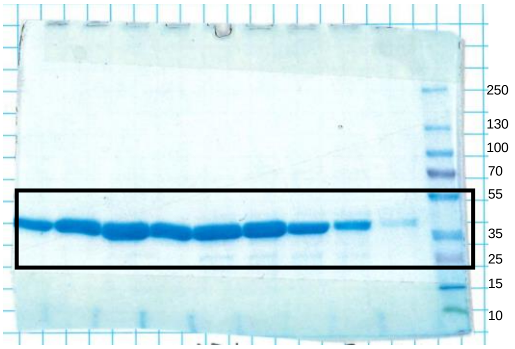

Extended Data Fig. 1b

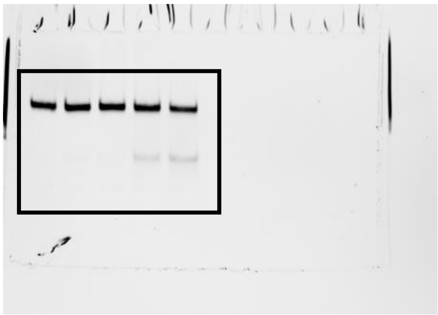

Extended Data Fig. 1c

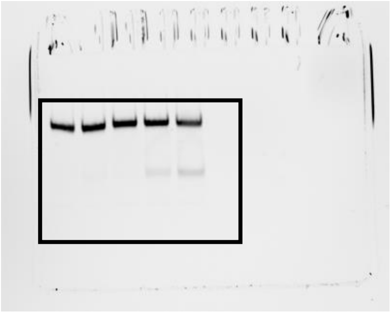

Extended Data Fig. 1d

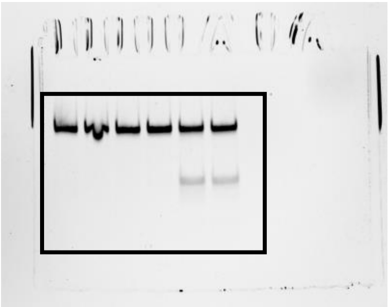

Extended Data Fig. 1e

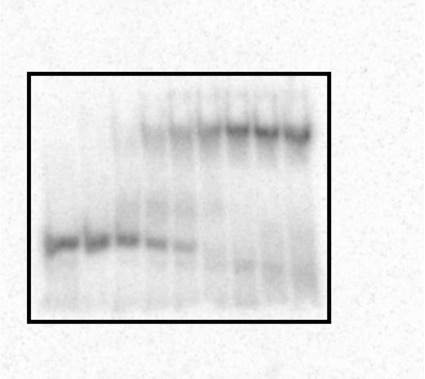

Extended Data Fig. 1f

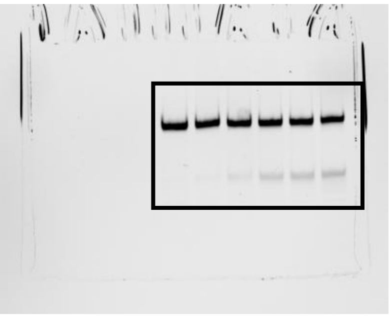

Fig. 1b (homo-)

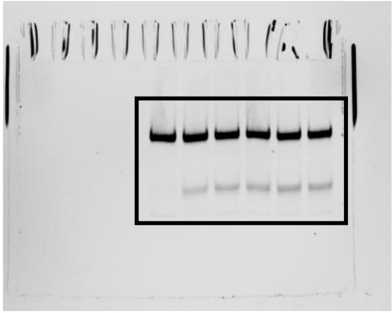

Fig. 1b (hetero-)

Supplementary information Table 1

**Residue and position**		**Location**	**Function**
S. thermophilus	*E. coli*		
Leu20	Ile23	L (*cis*-)	NTD coordination
Arg21	Arg24	L (*cis*-)	NTD coordination
Lys65	Lys68	L (*cis*-)	NTD coordination
Thr66	Thr69	L (*cis*-)	NTD coordination
Asp110	Asp113	L (*trans*-)	NTD coordination
Glu128	Glu131	L (*trans*-)	NTD coordination
Asp129	Asp132	L (*trans*-)	NTD coordination
Tyr131	Gln134	L (*trans*-)	NTD coordination
Arg171	Arg174	L (*trans*-)	NTD coordination
Thr193	Ser196	S (*cis*-)	NTD coordination
Ile196	Phe196	S (*cis*-)	NTD coordination
Phe197	Met200	S (*cis*-)	NTD coordination
Arg218	Arg221	S (*cis*-)	NTD coordination
Asn221	Asn224	S (*cis*-)	NTD coordination
Arg291	Arg294	H	DNA interaction
Pro310	Arg313	H	DNA interaction
Arg312	Pro315	H	DNA interaction
Arg315	Arg318	H	DNA interaction

Supplementary information Table 1. Key residues of the RuvB AAA+. To aid comparability with the heavily studied E. coli RuvAB-HJ system, the corresponding E. coli residues are denoted.

Supplementary information Table 2

```
S.thermophilus  MARILDNDLLGDEEYV---ERTLRPQYFKEYIGQDKVKDQLKIFIEAAKLRDEALDHTLL   57
E.coli          MIEADRLISAGTTLPEDVADRAIRPKLLEEYVGQPQVRSQMEIFIKAAKLRGDALDHLLI   60
S.typhimurium   MIEADRLISAGATIAEDVADRAIRPKLLAEYVGQPQVRSQMEIFIQAAKRRGDALDHLLI   60
                * .       *        :*::**: : **:** :*:.*::***:*** *.:**** *:

S.thermophilus  FGPPGLGKTTMAFVIANEMGVNLKQTSGPAIEKAGDLVAILNDLEPGDILFIDEIHRMPM   117
E.coli          FGPPGLGKTTLANIVANEMGVNLRTTSGPVLEKAGDLAAMLTNLEPHDVLFIDEIHRLSP   120
S.typhimurium   FGPPGLGKTTLANIVANEMGVNLRTTSGPVLEKAGDLAAMLTNLEPHDVLFIDEIHRLSP   120
                **********:* ::********: ****.:******.*:*.:*** *:********:

S.thermophilus  AVEEVLYSAMEDYYIDIMIGAGETSRSVHLDLPPFTLVGATTRAGMLSNPLRARFGINGH   177
E.coli          VVEEVLYPAMEDYQLDIMIGEGPAARSIKIDLPPFTLIGATTRAGSLTSPLRDRFGIVQR   180
S.typhimurium   VVEEVLYPAMEDYQLDIMIGEGPAARSIKIDLPPFTLIGATTRAGSLTSPLRDRFGIVQR   180
                .****** ***** :***** * ::**:::*******:******* *:.*** ****  :

S.thermophilus  MEYYELPDLTEIVERTSEIFEMTITPEAALELARRSRGTPRIANRLLKRVRDYAQIMGDG   237
E.coli          LEFYQVPDLQYIVSRSARFMGLEMSDDGALEVARRARGTPRIANRLLRRVRDFAEVKHDG   240
S.typhimurium   LEFYQVPDLQHIVGRSARHMGLEMSDDGALEVARRARGTPRIANRLLRRVRDFAEVKHDG   240
                :*:*::***  ** *::. : : :: :.***:***:***********:****:*::  **

S.thermophilus  VIDDKIADQALTMLDVDHEGLDYVDQKILRTMIEMYGGGPVGLGTLSVNIAEERETVEDM   297
E.coli          TISADIAAQALDMLNVDAEGFDYMDRKLLLAVIDKFFGGPVGLDNLAAAIGEERETIEDV   300
S.typhimurium   AISAEIAAQALDMLNVDAEGFDYMDRKLLLAVIDKFFGGPVGLDNLAAAIGEERETIEDV   300
                .*. .** *** **:** **:**:*:*:* ::*: : ******..*:. *.*****:**:

S.thermophilus  YEPYLIQKGFIMRTRTGRVATAKAYEHMGYDYTRDN                        333
E.coli          LEPYLIQQGFLQRTPRGRMATTRAWNHFGITPPEMP                        336
S.typhimurium   LEPYLIQQGFLQRTPRGRMATVRAWNHFGITPPEMP                        336
                 ******:**: **  **:**.:*::*:*    .
```

Supplementary information Table 2. **RuvB protein sequence alignment.**

Supplementary information Table 3

Nr.	Name	Sequence (5` - 3`)
1	wtRuvA_SB_1x - Fw	ATCTACGACCATGGTAGGCAGACTCAGAG
2	wtRuvA_SB_73ws1 Rw	GTGGCACCAGAGCGAGCTCTCAGTGATGGTGATGTAACGCGGC GCGTA
3	RuvB_Str_62b – Fw	ATCTAATGCCATGGTATACCCATACGATGTTCCAGATTACGCTGA GCGTACCCTTCGCCC
4	RuvB_Str_63 – Rw	AATTGTACAAGCTTTTAATTATCTCGAGTATAGTCATA
5	Hj-Y2ap_1	CTCATCGAGAATCTGCCGAGAGACCGAGCAGAATTCTATGTGTT TACCAAGCGCTG
6	Hj-Y2ap_2	CAGCGCTTGGTAAACACATAGAATTCTGCTCGGTCTGAGCCGTC TAAGAGACCTAG
7	Hj-Y2ap_3	CTAGGTCTCTTAGACGGCTCACTGGCTGTGGGATCCGAGCTGTC TAGAGACATCGA
8	Hj-Y2ap_4	TCGATGTCTCTAGACAGCTCGGATCCCACAGCCAGTCTCGGCAG ATTCTCGATGAG
9	HJ-X26	CCGCTACCAGTGATCACCAATGGATTGCTAGGACATCTTTGCCC ACCTGCAGGTTCACCC
10	HJ-X26	TGGGTGAACCTGCAGGTGGGCAAAGATGTCCTAGCAATCCATTG TCTATGACGTCAAGCT
11	HJ-X26	GAGCTTGACGTCATAGACAATGGATTGCTAGGACATCTTTGCCG TCTTGTCAATATCGGC
12	HJ-X26	TGCCGATATTGACAAGACGGCAAAGATGTCCTAGCAATCCATTG GTGATCACTGGTAGCGG

Supplementary information Table 3. **List of DNA primers used in this study.**

Video legends (1-6)

Video 1: RuvB motors rotate with respect to the RuvA core complex.

Two 3D classes of bipartite RuvAB-HJ particles are aligned to the RuvA core complex. Linear interpolation (Morph) of the two 3D classes reveals that the RuvB motor undergoes a rotational motion of ~60° with respect to the RuvA core complex. The rotation is evidenced by the position of the two $RuvA^{D3}$ (side view) and the DNA-binding RuvB subunits A, B, C and D, which, together, form the spiral staircase (top view). The data shown correspond to Extended Data Fig. 6.

Video 2: Bipartite RuvAB-HJ particles are highly flexible.

Shown are linear interpolations (Morphs) of maps corresponding to the seven most abundant eigenvectors (~95% of all motions), identified by principal component analysis using the multibody refinement tool in Relion[1]. Captions 1 and 2 show rotational motions (~45%), 3-6 show wobbling motions (~47.5%) and 7 shows a bouncing motion (~2.5%). The data shown correspond to Extended Data Fig. 7.

Video 3: $RuvA^{D3}$ binding exerts a wedge-like effect on the RuvB hexamer.

Video first shows an overview over the RuvB hexamer before zoning into the *converter* represented by RuvB subunits $F{:}E{:}D^{L}$. A linear interpolation between nucleotide cycle states s1 and s2 highlights the wedge-like effect that is exerted on to the RuvB hexamer through the binding of $RuvA^{D3}$ to RuvB subunit E. Arrows are only displayed for every other amino acid, and only if the distances are longer than 1 Å. For better representation, the length of the arrows is increased by a factor of 2.5.

Video 4:Motions associated with ATP hydrolysis in the RuvB hexamer.

Video first shows an overview over the RuvB hexamer before zoning into the ATP hydrolysis interface between RuvB subunits A and F. To illustrate the motion associated with ATP hydrolysis all five states have been aligned to RuvB subunit A. A linear interpolation between nucleotide cycle states s1 and s5 shows forward signalling (s1→s2) leading to ATP hydrolysis and retrograde signalling (s3→s5) leading to nucleotide exchange. Of note, forward signalling is triggered by the binding of $RuvA^{D3}$ (s1→s2). A magnification of the nucleotide binding pocket is shown in the upper left corner.

Video 5: Motions in RuvB subunits E and D associated with nucleotide exchange.

Video first shows an overview over the RuvB hexamer before zoning into the *converter* RuvB subunits $E{:}D^{L}$. To illustrate the motions associated with nucleotide exchange all five states have been aligned to the rigid part of RuvB subunit D [181-331]. A linear interpolation between nucleotide cycle states s1 to s5 shows the motions of subunit E and the large ATPase domain of subunit D associated with the escape of the ADP molecule in RuvB subunit D (s1→s2), transition through apo-like states (s2→s4) and the acquisition of a new ATP molecule (s4→s5). To aid visualization of these processes, the two $RuvA^{D3}$ are not shown. Also, the ADP nucleotide in RuvB subunit E (orange) has been omitted.

Video 6: Gate-keeping motions of the RuvB N-terminus associated with nucleotide exchange.

RuvB subunits E and D are superimposed. Video first shows linear interpolation (Morph) of RuvB subunit E as it progresses through nucleotide cycle states s1 to s5 of cluster [E]. RuvB subunit E then turns into subunit D (*cluster switch*) and the video shows progression of RuvB subunit D through nucleotide cycle states s1 to s5 of cluster [D]. The linear interpolation shows the gate-keeping motion of the RuvB N-terminus. First, the RuvB N-terminus containing cis-Leu20 and cis-Arg21 retracts from the ADP molecule in the nucleotide binding pocket of RuvB subunit E (orange). Retraction is completed when RuvB subunit E (orange) underwent a *cluster switch* and has turned into subunit D (beige). Upon acquisition of a new ATP molecule, the gate-keeping interactions between the RuvB N-terminus and the nucleotide are restored.

Video 7: Forward and retrograde signalling within the RuvB hexamer.

Video of consecutive conformational changes within the RuvB hexamer over five intermediate states of the nucleotide cycle (s1→→s5). For each transition, the initial structure of the respective state is shown in a surface representation and the direction of the conformational change is marked by arrows. Base and tip of the arrows start and end at the C-alpha coordinates of the initial and final structure of each transition. Arrows are only displayed for every other amino acid, and only if the distances are longer than 1 Å. For better representation, the length of the arrows is increased by a factor of 2.5. Immediately afterwards, the transition is also morphed in cartoon representation. Colors are used to distinguish among subunits. Major conformational changes during the nucleotide cycle are restricted to the converter, which is composed of subunits F, E, and D (large domain), and can be distinguished into a forward (s1→s2) and retrograde signalling wave (s3→s4→s5).

Video 8: Animation of the rotating RuvB motor lifting the DNA substrate.

RuvB motors processing a HJ are likely brought into rotation due to the advancing and rewinding substrate. As for lifting RuvB motors, the immobilization of the substrate-disengaged RuvB subunit in cluster [E] of the *converter* enables RuvB motors to transform the conformational changes associated with the nucleotide cycle into a lever action. This causes the DNA substrate to be lifted by 2 base pairs per ATP hydrolysis event. The conformational changes associated with the nucleotide cycle culminate in a *cluster switch,* which causes the conformational clusters [A-F] to progress around the ring and the RuvB motor to “walk back” on the lifted DNA substrate. As the sequential nucleotide cycle and the RuvB motor rotation progress in opposite directions, conformational clusters are maintained in a fixed position. The first three *cluster switches* are shown in grey colors to emphasize the fact that the structural changes associated with this event have not been observed structurally but are inferred from our five nucleotide cycle states. For simplicity, $RuvA^{D3}$ is not shown.

Video 9: Deconvolution I - Animation of the RuvB motor lifting the DNA substrate.

To deconvolute the processes shown in video 7, this video shows the action (lift) of a non-rotating RuvB motor in which RuvB subunit E (orange) is immobilized. For simplicity, immobilizing $RuvA^{D3}$ is not shown. RuvB motors perform sequential ATP hydrolysis events. Immobilization of the substrate-disengaged RuvB subunit in cluster [E] (orange) of the *converter* enables RuvB motors to transform the conformational changes associated with each nucleotide cycle into a lever action. This causes the DNA substrate to be lifted by 2 base pairs per nucleotide cycle (ATP hydrolysis event). The conformational changes associated with the nucleotide cycle culminate in a *cluster switch,* which causes the conformational clusters [A-F] to progress around the ring and the RuvB motor to “walk back” on the lifted DNA substrate. The first three *cluster switches* are shown in grey colors to emphasize the fact that the structural changes associated with this event have not been observed structurally but are inferred from our five nucleotide cycle states.

Video 10: Deconvolution II - Animation of the RuvB motor walking on the DNA substrate.

To deconvolute the processes shown in video 7, this video shows the action (walking) of a non-rotating RuvB motor in which RuvB subunit E is not immobilized. RuvB motors perform sequential ATP hydrolysis events. The conformational changes associated with each nucleotide cycle culminate in a *cluster switch*, which causes the conformational clusters [A-F] to progress around the ring. The *cluster switch* is inferred from our nucleotide cycle states but has not been resolved structurally. Therefore, we use grey colors to illustrate the conformational changes that are associated with the first three *cluster switches*. If the substrate-disengaged RuvB subunit in cluster [E] of the *converter* is not immobilized (i.e. $RuvA^{D3}$ is not bound), iterating through sequential nucleotide cycles and *cluster switches* causes the RuvB motor to walk on the DNA substrate. Of note, repositing (walking) of the RuvB motors on the DNA substrate through sequentially progressing nucleotide cycles inducing iterative *cluster switches* might represent the mechanistical basis for the previously described helicase activity of isolated RuvB motors.

Video 11: Animation of RuvAB-HJ complex assembly and processing of the HJ.

Two RuvA tetramers (green, cyan) assemble around the central HJ crossover. For the ‘inactive’ lower RuvA tetramer (green) only $RuvA^{D1}$ and $RuvA^{D2}$ complexing the HJ are shown. For the ‘processive’ upper RuvA tetramer (cyan), the linkers connecting $RuvA^{D2}$ to $RuvA^{D3}$ have been added to aid visualization of the branch migration process. Notably, the linkers are not resolved structurally and, therefore, their motions have been inferred. Two RuvB motors assemble around the DNA substrates leaving the RuvA-HJ complex, a process which is likely guided by $RuvA^{D3}$. RuvB motors primarily bind to one DNA strand (red). In processive mode (s1→s5), the four $RuvA^{D3}$ domains of a single tetramer (cyan) bind to the substrate-disengaged RuvB subunits E and D in the converters (purple colors) of the two RuvB motors. Binding of $RuvA^{D3}$ to RuvB subunit E (s1→s2) stimulates the nucleotide cycle and immobilizes the converter, allowing the conformational changes associated with the nucleotide cycle (s1→s5) to be transformed into a lever action. The translocation of the substrate causes its rewinding at the HJ crossover, which brings both RuvB motors into rotational motion. The conformational changes associated with the nucleotide cycle culminate in a *cluster switch,* which causes the conformational clusters [A-F] to progress around the ring and the RuvB motor to ‘walk back” on the lifted DNA substrate. As the sequential nucleotide cycle and the RuvB motor rotation progress in opposite directions, conformational clusters are maintained in a fixed position with respect to the RuvA core complex, enabling the release and recycling of the $RuvA^{D3}$ binding events. Notably, the release of $RuvA^{D3}$ from their *converters* has not been resolved structurally but has been animated to aid visualization of the branch migration process. Also, the branch migration process was animated under the assumption that the four transitions between the five states (s1→s5) correspond to four equal 15° rotations of the RuvB motor (4 transitions x 15° = 60° rotation).
