# Supplementary material for: Mechanism of AAA+ ATPase-mediated RuvAB–Holliday junction branch migration
Source: Nature. 2022 Aug 24;609(7927):630–9. doi: 10.1038/s41586-022-05121-1 (PMC9477746; doi:10.1038/s41586-022-05121-1)
Supplement: Supplementary file 2 — Reporting Summary [file 41586_2022_5121_MOESM2_ESM.pdf]

nature portfolio

Corresponding author(s): Jiri Wald, Dirk Fahrenkamp, Thomas C Marlovits

Last updated by author(s): 8.6.2022

Reporting Summary

Nature Portfolio wishes to improve the reproducibility of the work that we publish. This form provides structure for consistency and transparency in reporting. For further information on Nature Portfolio policies, see our Editorial Policies and the Editorial Policy Checklist.

Statistics

For all statistical analyses, confirm that the following items are present in the figure legend, table legend, main text, or Methods section.

n/a	Confirmed	
☐	☒	The exact sample size (*n*) for each experimental group/condition, given as a discrete number and unit of measurement
☒	☐	A statement on whether measurements were taken from distinct samples or whether the same sample was measured repeatedly
☒	☐	The statistical test(s) used AND whether they are one- or two-sided<br>*Only common tests should be described solely by name; describe more complex techniques in the Methods section.*
☒	☐	A description of all covariates tested
☒	☐	A description of any assumptions or corrections, such as tests of normality and adjustment for multiple comparisons
☒	☐	A full description of the statistical parameters including central tendency (e.g. means) or other basic estimates (e.g. regression coefficient) AND variation (e.g. standard deviation) or associated estimates of uncertainty (e.g. confidence intervals)
☒	☐	For null hypothesis testing, the test statistic (e.g. F, t, r) with confidence intervals, effect sizes, degrees of freedom and P value noted<br>*Give P values as exact values whenever suitable.*
☒	☐	For Bayesian analysis, information on the choice of priors and Markov chain Monte Carlo settings
☒	☐	For hierarchical and complex designs, identification of the appropriate level for tests and full reporting of outcomes
☒	☐	Estimates of effect sizes (e.g. Cohen's *d*, Pearson's *r*), indicating how they were calculated

Our web collection on statistics for biologists contains articles on many of the points above.

Software and code

Policy information about availability of computer code

Data collection	CryoEM: Thermo Fisher EPU v1.09 and v2.4, Negative stain EM: Thermo Fisher TIA v4.15
Data analysis	MotionCor2 v1.2.1 and v1.3, CTFfind v4.1.14, crYOLO v1.4, Gautomatch v0.56, RELION2, 3.1-beta and 3.1, Chimera v 1.11 and v1.13.1 and v1.14, ChimeraX v1.1 and 1.2.5, Pymol v2.4.1, Phyre2 (unversioned), Phenix v1.18.2, EMringer (unversioned), ISOLDE v1.0b5 and v1.1.2, MolProbity (unversioned), Phenix.real_space_refine v1.18-6831, Starmap v1.1.12, Rosetta v3.12

For manuscripts utilizing custom algorithms or software that are central to the research but not yet described in published literature, software must be made available to editors and reviewers. We strongly encourage code deposition in a community repository (e.g. GitHub). See the Nature Portfolio guidelines for submitting code & software for further information.

Data

Policy information about availability of data

All manuscripts must include a data availability statement. This statement should provide the following information, where applicable:

- Accession codes, unique identifiers, or web links for publicly available datasets
- A description of any restrictions on data availability
- For clinical datasets or third party data, please ensure that the statement adheres to our policy

Cryo-EM density maps resolved in this study have been deposited in the Electron Microscopy Data Bank (EMDB) (www.emdataresource.org) under accession codes: EMD-13294, EMD-13295, EMD-13296, EMD-13297, EMD-13298, EMD-13299, EMD-13300, EMD-13301, EMD-13302, EMD-13303, EMD-13304, EMD-13305,

EMD-15126 and EMD-15085. The corresponding coordinates have been deposited in the Protein Data Bank (PDB) (https://www.pdb.org) under accession codes: 7PBL, 7PBM, 7PBN, 7PBO, 7PBP, 7PBQ, 7PBR, 7PBS, 7PBT and 7PBU. Uncropped versions of all gels and blots are provided in Supplementary Fig. 2. All other data are available from the corresponding authors upon reasonable request.

Human research participants

Policy information about studies involving human research participants and Sex and Gender in Research.

Reporting on sex and gender	Not relevant to this study
Population characteristics	Not relevant to this study
Recruitment	Not relevant to this study
Ethics oversight	Not relevant to this study

Note that full information on the approval of the study protocol must also be provided in the manuscript.

Field-specific reporting

Please select the one below that is the best fit for your research. If you are not sure, read the appropriate sections before making your selection.

☒ Life sciences ☐ Behavioural & social sciences ☐ Ecological, evolutionary & environmental sciences

For a reference copy of the document with all sections, see nature.com/documents/nr-reporting-summary-flat.pdf

Life sciences study design

All studies must disclose on these points even when the disclosure is negative.

Sample size	Sample sizes were chosen as a maximum possible while considering practical limitations for data collection and subsequent data processing. The size of the final particle set was determined by the ability to reach resolutions sufficient in 3D reconstructions.
Data exclusions	No data was excluded from the analysis. During cryoEM data clustering, good cryoEM images were chosen for further processing based on their achieved resolution and 3D map quality, a standard method for cryoEM high resolution structural determination.
Replication	All biochemical and vitrification experiments have been successfully replicated.
Randomization	Particles/images were randomly partitioned for resolution and quality assessment.
Blinding	Blinding during data collection and analysis is not a commonly applied procedure in cryoEM.

Reporting for specific materials, systems and methods

We require information from authors about some types of materials, experimental systems and methods used in many studies. Here, indicate whether each material, system or method listed is relevant to your study. If you are not sure if a list item applies to your research, read the appropriate section before selecting a response.

Materials & experimental systems

n/a	Involved in the study	
☒	☐	Antibodies
☒	☐	Eukaryotic cell lines
☒	☐	Palaeontology and archaeology
☒	☐	Animals and other organisms
☒	☐	Clinical data
☒	☐	Dual use research of concern

Methods

n/a	Involved in the study	
☒	☐	ChIP-seq
☒	☐	Flow cytometry
☒	☐	MRI-based neuroimaging
